# Differential Deleterious Impact of Highly Saturated Versus Monounsaturated Fat Intake on Vascular Function, Structure, and Mechanics in Mice

**DOI:** 10.3390/nu13031003

**Published:** 2021-03-19

**Authors:** Elena Vega-Martín, Marta Gil-Ortega, Raquel González-Blázquez, Sara Benedito, Jesús Fernández-Felipe, Mariano Ruiz-Gayo, Nuria del Olmo, Julie A. Chowen, Laura M. Frago, Beatriz Somoza, María S. Fernández-Alfonso

**Affiliations:** 1 Instituto Pluridisciplinar, Universidad Complutense de Madrid, 28040 Madrid, Spain; elevega@ucm.es (E.V.-M.); marisolf@farm.ucm.es (M.S.F.-A.); 2 Departamento de Farmacología, Farmacognosia y Botánica, Facultad de Farmacia, Universidad Complutense de Madrid, 28040 Madrid, Spain; 3 Departamento de Ciencias Farmacéuticas y de la Salud, Facultad de Farmacia, Universidad San Pablo-CEU, CEU Universities, 28925 Madrid, Spain; raquelgonzalezblazquez@gmail.com (R.G.-B.); jesus.fernandezfelipe@beca.ceu.es (J.F.-F.); ruigayo@ceu.es (M.R.-G.); nuriadelolmo@psi.uned.es (N.d.O.); bsomoza.fcex@ceu.es (B.S.); 4 Departamento de Fisiología, Facultad de Farmacia, Universidad Complutense de Madrid, 28040 Madrid, Spain; sbenedi@farm.ucm.es; 5 Department of Psychobiology, School of Psychology, National Distance Education University (UNED), 28040 Madrid, Spain; 6 Department of Pediatric Endocrinology, Hospital Infantil Universitario Niño Jesús, Instituto de Investigación la Princesa, Centro de Investigación Biomédica en Red Fisiopatología de la Obesidad y la Nutrición (CIBEROBN), Instituto de Salud Carlos III, 28009 Madrid, Spain; julieann.chowen@salud.madrid.org (J.A.C.); laura.frago@uam.es (L.M.F.); 7 IMDEA Food Institute, CEI UAM + CSIC, 28049 Madrid, Spain; 8 Department of Pediatrics, Facultad de Medicina, Universidad Autónoma de Madrid, 28029 Madrid, Spain; 9 Instituto de Investigación Sanitaria San Carlos (IdiSSC), 28040 Madrid, Spain

**Keywords:** saturated fatty acids, monounsaturated fatty acids, purified high-fat diets, endothelial dysfunction, nitric oxide, vascular remodeling, arterial stiffness, collagen

## Abstract

Vegetable oils such as palm oil (enriched in saturated fatty acids, SFA) and high-oleic-acid sunflower oil (HOSO, containing mainly monounsaturated fatty acids, MUFA) have emerged as the most common replacements for trans-fats in the food industry. The aim of this study is to analyze the impact of SFA and MUFA-enriched high-fat (HF) diets on endothelial function, vascular remodeling, and arterial stiffness compared to commercial HF diets. Five-week-old male C57BL6J mice were fed a standard (SD), a HF diet enriched with SFA (saturated oil-enriched Food, SOLF), a HF diet enriched with MUFA (unsaturated oil-enriched Food, UOLF), or a commercial HF diet for 8 weeks. Vascular function was analyzed in the thoracic aorta. Structural and mechanical parameters were assessed in mesenteric arteries by pressure myography. SOLF, UOLF, and HF diet reduced contractile responses to phenylephrine and induced endothelial dysfunction in the thoracic aorta. A significant increase in the β-index, and thus in arterial stiffness, was also detected in mesenteric arteries from the three HF groups, due to enhanced deposition of collagen in the vascular wall. SOLF also induced hypotrophic inward remodeling. In conclusion, these data demonstrate a deleterious effect of HF feeding on obesity-related vascular alterations that is exacerbated by SFA.

## 1. Introduction

Obesity constitutes one of the major preventable risk factors for the development of several noncommunicable diseases including cardiovascular alterations, diabetes, musculoskeletal disorders, and some cancers [1]. In fact, in both humans and mice models, increasing evidence has shown that obesity favors the development of vascular damage, such as endothelial dysfunction, that seems to be due, at least in part, to compromised nitric oxide (NO) availability [2,3] and/or increased oxidative stress [3,4]. In addition to endothelial dysfunction, the two other crucial mechanisms implicated in vascular alterations are arterial remodeling and stiffness. Indeed, chronic alterations in vascular structure may lead to significant changes in mechanical properties, such as compliance and distensibility [5], thus accounting for arterial stiffness, which has recently emerged as an independent risk factor for cardiovascular diseases [6,7]. Conversely, weight loss in overweight and obese individuals is associated with a reduction in arterial stiffness [8]. In addition, we have recently demonstrated that extracellular matrix remodeling, including an increase in collagen content and elastin fragmentation, plays a key role in the development of central arterial stiffness due to obesity [7].

For the last decades, the most common strategy to induce obesity in animal models has been the use of commercial high fat (HF) diets providing between 45 and 65% of energy from fat. However, despite being widely used, the purified commercial diets exhibit important nutritional differences compared to standard chow diets. In fact, whereas the main source of fat in chow diets is vegetable and fish oil, purified HF diets mainly contain lard, especially rich in saturated fatty acids (SFA) but very limited in monounsaturated (MUFA) and polyunsaturated fatty acids (PUFA). In addition, purified diets also provide a greater amount of easily metabolizable carbohydrates as compared to standard chow diets for rodents [9,10]. Therefore, commercial HF diets might not be fully representative of common diets ingested by the general population worldwide.

Despite the limited evidence concerning the specific impact of different fatty acids on vascular alterations, several studies performed on obese humans have shown that HF diets enriched in SFA impair flow-mediated dilation or endothelial function [11,12]. Similarly, a potential negative impact of SFA-enriched diets, but not of MUFA-enriched diets, on arterial stiffness has been also suggested [13]. However, a study performed by Sanders et al. [14] failed to show a beneficial effect of replacing SFA-enriched diets with MUFA-enriched diets elaborated from refined high-oleic-acid sunflower oil (HOSO) on endothelial function or arterial stiffness [14]. Contrarily, a study performed in spontaneously hypertensive rats evidenced a significant improvement in endothelial function in rats fed a MUFA-enriched diet elaborated from virgin olive oil [15].

Because the use of vegetable oils such as palm oil and HOSO has dramatically increased in food industry in the past decades in an attempt to replace trans fats [16], elucidating the precise effect of these oils on vascular alterations is of utmost importance. In this context, we hypothesized that HF diets enriched in SFA are more harmful than HF diets enriched in MUFA on the development of vascular alterations derived from obesity. Therefore, the main aim of this study was to analyze the differential impact of a SFA-enriched HF diet and a MUFA-enriched HF diet on endothelial function, vascular remodeling and the development of arterial stiffness as compared to commercial standard HF diets, as well as to characterize the mechanisms involved in these alterations.

## 2. Materials and Methods

### 2.1. Animals and Experimental Protocol

Four-week-old male C57BL/6J mice (Charles River, Écully, France) were housed under controlled dark-light cycles (12 h/12 h) and temperature (22 °C) and had access to food and water ad libitum. After one week of acclimation, animals were randomly divided into four groups (n = 7–10) with a similar average body weight (BW) and assigned to a standard diet (SD, 18% energy from fat; Harlan Laboratories, España, Spain), a HF diet enriched in saturated fat (saturated oil-enriched Food, SOLF, 70% energy from fat), a HF diet enriched in monounsaturated fat (unsaturated oil-enriched Food, UOLF, 70% energy from fat), or a commercially available high-fat diet (HF, 62% energy from fat, Test Diets, UK) for 8 weeks. UOLF and SOLF diets were elaborated by mixing standard chow diet (60%) and 40% of either HOSO or palm kernel oil, respectively, as previously described [17]. BW and food intake were monitored weekly. Animals were euthanized and exsanguinated by decapitation. The thoracic aorta, the superior mesenteric artery and mesenteric resistance arteries were immediately dissected and used for vascular studies. Blood was collected in EDTA-coated tubes, centrifuged at 800× *g* for 10 min and plasma samples were stored at −80 °C until used for biochemical analysis.

The Institutional Animal Care and Use Committee approved all experimental procedures according with the European Union Laboratory Animal Care Rules (86/609/EEC) and were approved by the Animal Research Committee of San Pablo CEU University (PCD-CEU08-112-16 and PROEX 200/18). All efforts were made to avoid animal suffering in accordance with the ARRIVE guidelines for reporting experiments involving animals [18,19]. All experimental procedures were blinded.

### 2.2. Assessment of Biochemical Parameters

Glucose was assessed by a spectrophotometric method (Glucose Trinder Method, Roche Applied Science, Penzberg, Spain). Triglycerides and non-esterified fatty acids were analyzed using the GPO (Biolabo, Maizy, France) and the ACS-ACOD (Wako, Bioproducts, Germany) methods, respectively.

### 2.3. Functional Studies in the Thoracic Aorta Artery

The thoracic aorta was carefully isolated and placed in oxygenated, cold physiological Krebs Henseleit buffer (KH, 115 mM NaCl, 2.5 mM CaCl_2,_ 4.6 mM KCl, 25 mM NaHCO_3_, 1.2 mM MgSO_4_, 1.2 mM KH_2_PO_4_, 0.01 mM EDTA, and 11.1 mM glucose), deprived of perivascular adipose tissue and blood and cut into rings of 2–3 mm length. Vascular rings were then suspended around two intraluminal parallel wires and placed into an organ bath containing KH at pH = 7.4, 37 °C and bubbled with carbogen (95% O_2_–5% CO_2_) and connected to a force transducer. Isometric tension was recorded in a Power Lab system (AD Instruments, Oxford, UK). An optimal resting tension of 1 g was applied to aortic rings and was readjusted every 10 min. After 40 min period of equilibration, arterial contractility was assessed using a potassium chloride solution (KCl, 60 mM). Cumulative concentration-response curves in response to phenylephrine (Phe, 10^−8^–10^−6^ M) were performed. Relaxation curves in response to acetylcholine (Ach, 10^−9^–10^−4^ M) were also carried out in segments pre-contracted with Phe (from 10^−6^ to 10^−5^ M, as required to ensure a similar pre-contraction in all groups). The nitric oxide synthase inhibitor, NG-nitro-L-arginine methyl ester (L-NAME, 10^−4^ M), was added and the tissue was incubated 30 min prior to the addition of Phe. All reagents were provided by Sigma-Aldrich (Madrid, Spain).

### 2.4. Structural and Mechanical Properties in Mesenteric Resistance Arteries

Structural and mechanical properties were assessed in first-order branch mesenteric resistance arteries (MRA) by pressure myography (Model P100, Danish Myo-Tech, Hinnerup, Denmark), as previously described [20]. Briefly, intraluminal pressure was set at 70 mmHg for 30 min to stabilize MRA segments, which were incubated in calcium-free KH (0 Ca^2+^-KH; 115 mM NaCl, 25 mM NaHCO_3_, 4.6 mM KCl, 1.2 mM MgSO_4_, 1.2 mM KH_2_PO_4_, 10 mM EGTA and 5.5 mM glucose) at 37 °C and bubbled with carbogen. External and internal diameters (D_i0Ca_ and D_e0Ca_, respectively) were measured at increasing intraluminal pressures (5, 20, 40, 60, 80, 100, 120, and 140 mmHg). Thereafter, MRA segments were fixed with 4% paraformaldehyde (in 0.2 M phosphate buffer, pH 7.2–7.4) at 70 mmHg and 37 °C for 45 min and stored at 4 °C for confocal microscopy studies. Structural (lumen and vessel diameters, wall thickness, cross-sectional area (CSA) and wall-to-lumen ratio) and mechanical parameters (stress, strain, and incremental distensibility) were calculated from D_e0Ca_ and D_i0Ca_ values as previously described [21]. Arterial stiffness was assessed by the parameter β, the slope of the stress–strain relationship and a measure of intrinsic arterial stiffness [22].

### 2.5. Elastin Content and Organization in Mesenteric Resistance Arteries

Elastin content and organization were determined in the external (EEL) and internal elastic laminae (IEL) of previously fixed MRA by fluorescent confocal microscopy based on the auto fluorescent properties of elastin (excitation 488 nm/emission 500–560 nm). To avoid artery deformation, intact arterial segments were mounted with antifading solution (Citifluor) on a slide provided with a small well. MRA segments were visualized with a Leica TCS SP5 confocal microscope (Leica Microsystems, Wetzlar, Germany). Serial optical sections (stacks of images) from the adventitia to the lumen were captured with a 63× oil immersion objective at a wavelength of 488/515 nm. All images were captured under identical conditions of laser intensity, contrast, and brightness. Quantitative analyses were performed in three randomly selected regions of EEL and IEL of at least five independent experiments using Image J software [21]. From each stack of serial images, individual projections of IEL were reconstructed to measure total fenestra number and area. Elastin content was quantified from the mean fluorescence intensity values [23].

### 2.6. Collagen Content in Superior Mesenteric Arteries

Superior mesenteric arteries were homogenized in a lysis buffer containing (0.5 M NaCl, 0.1 M Na_4_P_2_O_7_, 0.5 M dichloro diphenyl-trichloroethane, 0.5 M HEPES, 0.5 M NaF, 0.5 M Na_3_VO_4_, 0.1 M EDTA, 0.1 M EGTA, 20% glycerol, 0.2 M PMSF, 1 μL/mL leupeptin, 1 μL/mL N-α-p-tosyl-1-lysine in chloromethylketone [TLCK] and 1 μL/mL aprotinin) using a Tissue Lyser homogenizer (Qiagen, Hilden, Germany) and applying four cycles at 50 Hz for 5 min. Samples were subjected to thermal shocks (3x 37 °C/liquid nitrogen) and centrifuged at 10,000 rpm and 4 °C for 10 min. Supernatants were collected and protein concentration was assessed with the method described by Bradford [24].

Collagen content was quantified using a dot-blot-Sirius red-based assay, as previously described [25]. Briefly, 2 μL of samples were applied to PVDF membranes (BioRad, Spain), that were subsequently dried at 37 °C for 5 min to favor sample fixation and to reduce nonspecific binding of Sirius red (Sigma-Aldrich, Tres Cantos, Spain) to PVDF membranes. Membranes were incubated in 2.5 × 10^−3^% *w*/*v* Sirius red dissolved in saturated picric acid for 30 min at 4 °C, washed in distilled water three times for 1 min and scanned with a Chemi Doc System (ChemiDoc XRS+ Imaging System BioRad, Alcobendas, Spain). Collagen staining was quantified using Image Lab 3.0 software (BioRad). Results were interpolated in a calibration curve (0.1 to 6 μg/μL) using gelatin from porcine skin, Type A and gel strength 300 (Sigma-Aldrich, Spain).

### 2.7. Statistical Analysis

Contractile responses to Phe are expressed in absolute values. Relaxation to Ach is expressed as the percentage of the previous contractile response to Phe. The maximal response (E_max_ values) and the potency (pD_2_ values) were calculated by using nonlinear regression analyses of each individual concentration-response curve. The area under the curve (AUC) was determined from each individual concentration-response curve plot. All values are given as mean ± S.E.M. and n denotes the number of replicates used in each experiment. Student’s *t* tests or ANOVA followed by Bonferroni or Tukey post-hoc test was used as appropriate. A value of *p* < 0.05 was considered statistically significant. Statistical analysis was performed using GraphPad Prism 7.0 (GraphPad Software, San Diego, CA, USA).

## 3. Results

### 3.1. SOLF, UOLF, and HF Diets Increase Body Weight and Glucose Levels But Differently Affect NEFA Concentrations

**Figure 1:**
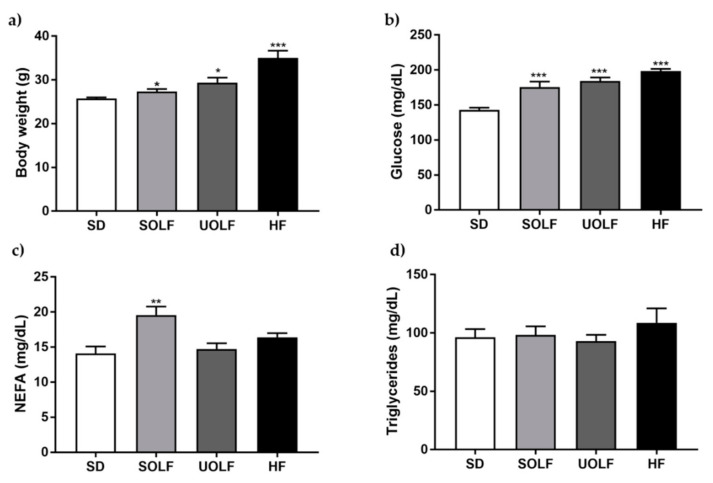
Body weight and biochemical parameters. Bar graphs showing body weight (**a**) and plasmatic concentrations of glucose (**b**), non-esterified free-fatty acids (NEFA); (**c**) and triglycerides (**d**). Data are shown as mean ± SEM of 7–10 animals per strain. * *p* < 0.05, ** *p* < 0.01, *** *p* < 0.001 vs. the control group SD.

BW was significantly higher in SOLF, UOLF, and HF mice compared to the SD group (Figure 1a). However, the increase in BW was more pronounced in UOLF (14%) and especially in HF mice (36%) compared to SOLF mice (6%) (*p* < 0.001, BW_SOLF_ vs. BW_HF_). In addition, whereas SOLF increased both glucose and NEFA plasma concentrations, the UOLF and HD diet only increased glycemia (Figure 1b,c). The fat-enriched diets did not modify triglyceride levels (Figure 1d).

### 3.2. SOLF, UOLF, and HF Diets Induce Endothelial Dysfunction and Reduce Contractile Responses to Phenylephrine in the Thoracic Aorta

**Figure 2:**
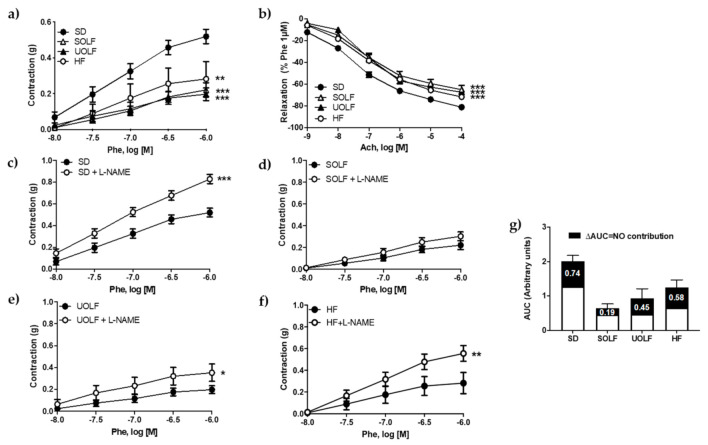
Characterization of vascular function. Concentration–response curves to (**a**) phenylephrine (Phe, 10^−8^ to 10^−6^ M) and (**b**) acetylcholine (Ach, 10^−9^ to 10^−4^ M) in aorta segments from standard (SD), unsaturated oil-enriched Food (UOLF), saturated oil-enriched Food (SOLF), and high-fat (HF) mice. Contractions are expressed in absolute values and relaxant responses are expressed as percentage of a previous contraction to Phe (10^−6^ M). Concentration–response curves to Phe (10^−8^ to 10^−6^ M) in aorta segments from SD (**c**), SOLF (**d**), UOLF (**e**), and HF mice (**f**) in absence and presence of L-NAME. (**g**) AUC in response to Phe in presence (full histogram) or absence (white bar) of L-NAME. The difference in AUC (black bar) represents NO bioavailability. Data are shown as mean ± SEM of 7–10 animals per group. * *p* < 0.05, ** *p* < 0.01, *** *p* < 0.001 vs. its corresponding control.

Maximal contractions to KCl (60 mM) were significantly reduced in aortic rings from both SOLF and UOLF compared to SD (SD = 0.57 ± 0.02 g, SOLF = 0.38 ± 0.02 g and UOLF = 0.40 ± 0.02 g, *p* < 0.001), with no changes detected in the HF group (0.58 ± 0.03 g). Contractile responses to Phe (10^−8^-10^−6^ M) were significantly diminished in arteries from SOLF, UOLF and HF animals compared to SD mice (Figure 2a), as evidenced by the maximal responses (E_max_SD = 0.52 ± 0.04 g, E_max_SOLF = 0.22 ± 0.04 g, E_max_UOLF = 0.20 ± 0.04 g; E_max_HF = 0.28 ± 0.1 g; *p* < 0.05 SOLF and HF and *p* < 0.01 UOLF *vs* SD). However, Phe potency was not modified by the diets (pD_2_SD = 7.02 ± 0.06; pD_2_SOLF = 6.89 ± 0.12; pD_2_UOLF = 6.99 ± 0.13; pD_2_HF = 6.92 ± 0.12). In addition, the functional integrity of the endothelium, as assessed by concentration-response curves to Ach (10^−9^ to 10^−4^ M), was also compromised in aortic rings from SOLF, UOLF, and HF mice as compared to the SD group (Figure 2b).

To determine whether these alterations were due to changes in NO contribution, basal NO availability was analyzed from the difference between response curves to Phe performed in the presence and absence of L-NAME (10^−4^ M). Pre-incubation with L-NAME significantly enhanced contractile responses to Phe in aortic rings from SD (Figure 2c), UOLF (Figure 2e) and HF (Figure 2f) mice. In contrast, L-NAME did not modify contractile responses to Phe in SOLF mice (Figure 2d). Therefore, and as evidenced by the difference between the AUC (Figure 2g), these data reveal that NO availability was significantly compromised by SOLF and to a lesser extent by UOLF, thus contributing to impair vascular functionality.

### 3.3. SOLF but Not UOLF or HF Diets Induced Hypotrophic Inward Remodeling in Mesenteric Resistance Arteries

**Figure 3:**
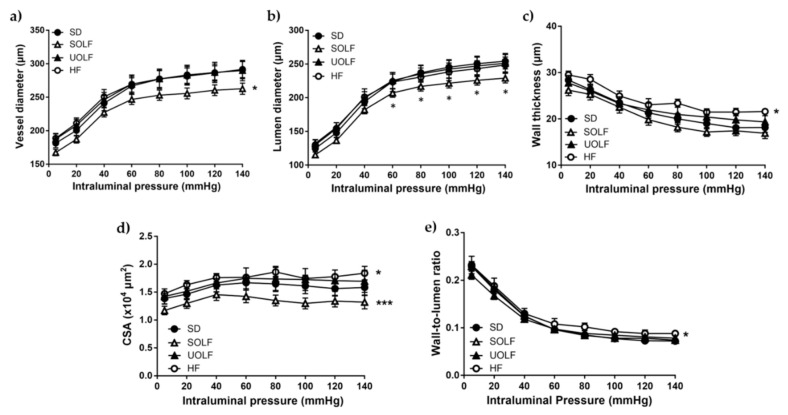
Characterization of structural parameters in first order mesenteric resistance arteries. (**a**) Vessel diameter–pressure, (**b**) lumen diameter–pressure, (**c**) wall thickness-pressure, (**d**) cross sectional area (CSA) pressure, and (**e**) media to lumen ratio-pressure of mesenteric resistance arteries (MRA) from SD, UOLF, SOLF, and HF mice. Results are expressed as mean ± SEM of n = 7–10. * *p* < 0.05 and *** *p* < 0.001 vs. the SD group.

The study of structural parameters revealed a significant reduction in the lumen diameter and CSA and, consequently, in the vessel diameter in MRA from SOLF mice compared to the SD without changes in the wall thickness and wall-to-lumen ratio (Figure 3a–e), thus demonstrating the development of hypotrophic inward remodeling. In contrast, HF mice exhibited a significant, though very mild, hypertrophic remodeling as evidenced by the increase in both the wall thickness and CSA together with a reduction in the wall-to-lumen ratio. Nevertheless, these alterations were not paralleled with changes either in the vessel or in the lumen diameter of HF mice’ MRA. The UOLF diet did not exert any modification in MRA’s structural parameters.

### 3.4. SOLF, UOLF, and HF Diets Induced Arterial Stiffness in Mesenteric Resistance Arteries

**Figure 4:**
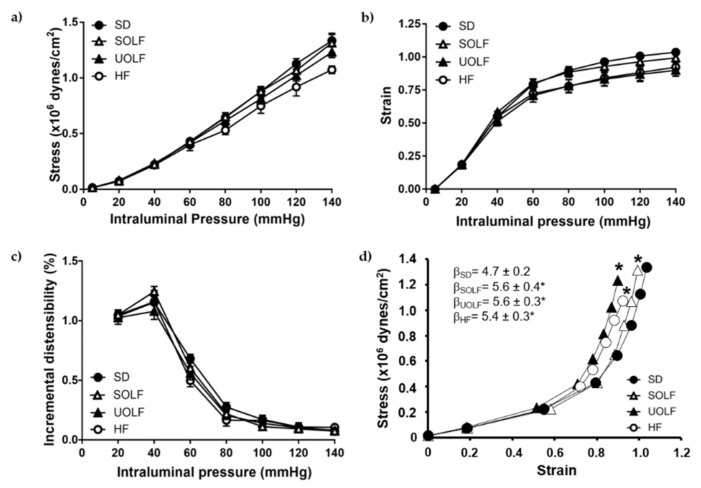
Characterization of mechanical parameters in first-order mesenteric resistance arteries. (**a**) Wall stress-pressure, (**b**) strain-pressure, (**c**) incremental distensibility-pressure curves and (**d**) stress-strain relationships with β values of MRA from SD, UOLF, SOLF, and HF mice. Results are expressed as mean ± SEM of n = 7–10. * *p* < 0.05 vs. the SD group.

The study of mechanical parameters showed no significant alterations in stress (Figure 4a), strain (Figure 4b), or incremental distensibility (Figure 4c) from MRA, when analyzed independently. However, the stress/strain relationship was significantly shifted to the left in MRA from SOLF, UOLF, and HF mice compared to the SD group. In addition, MRA from SOLF, UOLF, and HF mice exhibited a significant increase in β-index as compared to the SD group, suggesting increased intrinsic arterial stiffness in these arteries (Figure 4d).

### 3.5. SOLF, UOLF, and HF Diets Induced An Increase in Collagen Deposition but No Major Changes in Elastin Content or Organization

**Figure 5:**
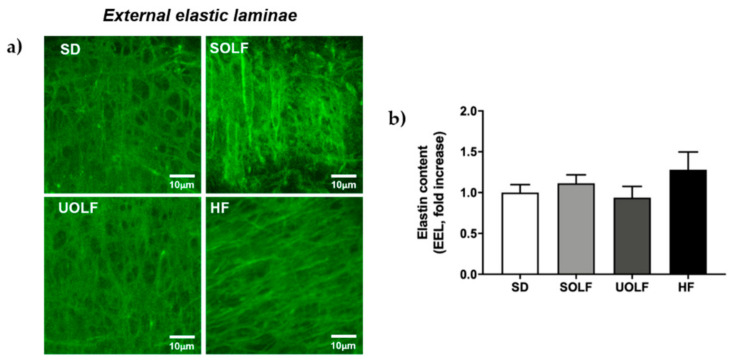
Elastin content and organization of external elastic laminae (EEL) in first-order MRA (**a**) Representative confocal microscopy images of projections of MRA’s EEL from SD, UOLF, SOLF, and HF mice. Projections were obtained from serial optical sections captured with a fluorescence confocal microscope (x63 oil immersion objective, zoom 2). (**b**) Bar graphs show fluorescence intensity, indicative of elastin content in EEL from SD, UOLF, SOLF, and HF animals. Results are expressed as mean ± SEM of n = 4–10.

**Figure 6:**
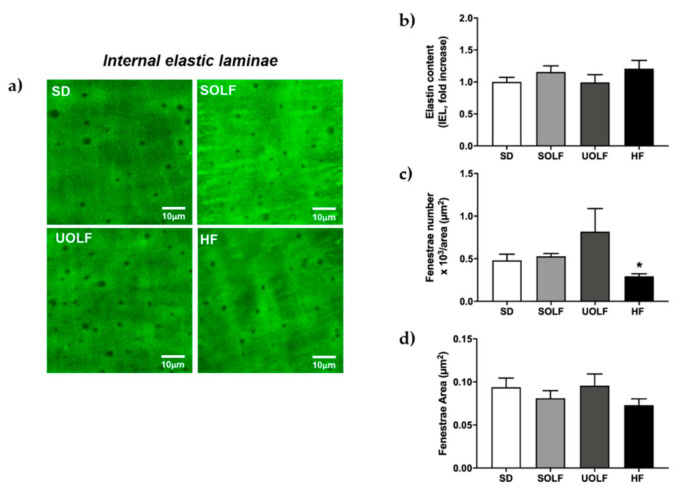
Elastin content and organization of internal elastic laminae (IEL) in first-order MRA. (**a**) Representative confocal microscopy images of projections of MRA’s IEL from SD, UOLF, SOLF, and HF mice. Projections were obtained from serial optical sections captured with a fluorescence confocal microscope (x63 oil immersion objective, zoom 2). Bar graphs show (**b**) fluorescence intensity, indicative of elastin content in the IEL, (**c**) fenestrae number, and (**d**) fenestrae area in MRA from SD, UOLF, SOLF, and HF animals. Results are expressed as mean ± SEM of n = 4–10. * *p* < 0.05 vs. the SD group.

To determine the mechanisms involved in the development of arterial stiffness, we assessed the impact of fat-enriched diets on the two major proteins regulating arterial distensibility, elastin, and collagen. Elastin content was not modified by the diets either in the external (EEL; Figure 5a,b) or in the internal elastic lamina (IEL; Figure 6a,b) from MRA. However, whereas SOLF and UOLF did not affect the fenestrae area or its number in the IEL, the HF diet significantly reduced the fenestrae number as compared to the SD group (Figure 6a,c), with no changes in the fenestrae area (Figure 6a,d).

**Figure 7:**
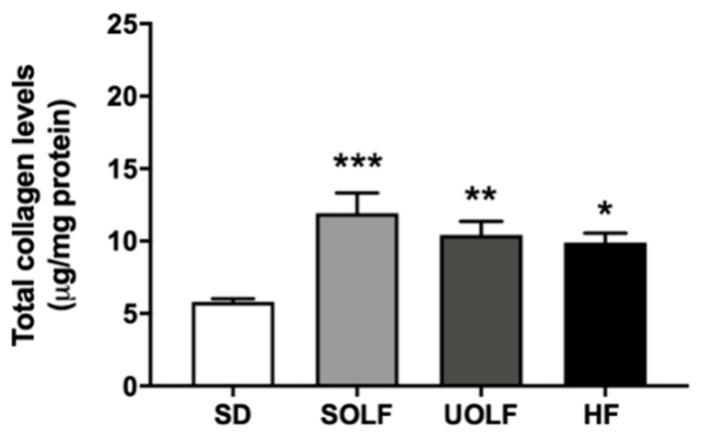
Collagen content in superior mesenteric arteries. Collagen content in the superior mesenteric artery from SD, UOLF, SOLF and HF mice. Results are expressed as mean ± SEM of n = 6. * *p* < 0.05, ** *p* < 0.01, *** *p* < 0.001 vs. the SD group.

In contrast, MRA from SOLF, UOLF, and HF mice exhibited higher amounts of collagen than the arteries from SD mice (Figure 7). Together, these results suggest that arterial stiffness induced by fat-enriched diets results from enhanced collagen deposition in the vascular wall.

## 4. Discussion

Numerous studies performed in mice/rat models of diet-induced obesity (DIO) [3,26,27,28] as well as in humans with obesity [29,30,31] have demonstrated a link between excessive energy intake and the development of endothelial dysfunction and arterial stiffness [7,32,33,34,35,36]. However, though most of these alterations have been generally attributed to an excessive intake of SFA [11,13,37,38], the net contribution of different fatty acid types to obesity-derived vascular alterations remains to be clarified. In this context, the novel findings of this study are that UOLF (enriched in vegetal-derived MUFA), as well as SOLF and HF diets (enriched in SFA from vegetal and animal sources, respectively) impair endothelial function and increase arterial stiffness as a result of enhanced collagen deposition in the arterial wall. SOLF also favors the development of hypotrophic inward remodeling.

As expected, the three fat-enriched diets induced an increase in BW as the result of an enhanced energy intake, as previously described [17]. However, BW increase was more evident in the HF group, what might be due to the fact that purified HF diets contain easily metabolizable carbohydrates, which might exacerbate weight gain [9]. In addition, and despite glucose levels being enhanced by SOLF, UOLF, and HF diet, only the SOLF group exhibited alterations in plasmatic concentrations of NEFA. It is important to note that a role for elevated NEFA on the development of endothelial dysfunction [2,27] and defective NO release [39] has been clearly demonstrated. In addition, a study performed in obese hypertensive patients revealed that elevated NEFA levels in plasma might also contribute to vascular growth and remodeling [40]. Therefore, the increase in NEFA observed exclusively in SOLF animals could contribute to the differential impact of SOLF vs. UOLF and HF diet on vascular alterations.

One of the major findings of this study is that UOLF, and not only SOLF and HF diet, impairs endothelial function after 8 weeks of diet. This result supports other studies showing a deleterious effect of SFA-enriched diets [37], manufactured either from animal (mainly lard) [3,26,27] or vegetable sources [13], on endothelial function. However, endothelial dysfunction displayed by UOLF animals was surprising, since a beneficial effect of olive oil-enriched diets has been reported by Herrera et al. [15]. It should be highlighted that this study also reported that the beneficial effects of olive oil were not observed in mice that consumed a HOSO-enriched diet, suggesting that the effect of olive oil is not linked to MUFA but to other olive oil components, like polyphenols, which are absent in HOSO [15]. Therefore, our current data suggest that the intake of elevated amounts of fat exerts a deleterious impact on vascular function, independently of fatty acid composition.

Another interesting finding in the current study is the significant reduction of contractile responses to Phe detected in SOLF, UOLF, and HF mice, which has been previously described in mice fed a 62% fat HF diet for only 4 weeks [26]. This could be due to alterations in alpha-1 receptor function, as suggested by Juarez et al. [41], though further studies are required to better address this matter. In contrast, a commercial diet providing 45% energy content from fat was not able to alter NA-induced contractions after even 32 weeks of dietary treatment [3], which evidences that the diets used in our study (which provide 62–70% energy from fat) are much more aggressive than 45% commercial HF diets.

We also analyzed the contribution of NO to vascular responses. As expected, NO bioavailability was significantly compromised in SOLF mice. However, this effect was less pronounced in either HF or UOLF mice. These data point to the existence of additional mechanisms accounting for endothelial dysfunction, different from a reduction in NO release, that must take place in HF and UOLF mice. Unfortunately, the poor contractile response to Phe, together with the rapid desensitization to Phe detected in SOLF, UOLF, and HF mice, hindered a further characterization of such mechanisms. In any case, a role of contractile prostaglandins or oxidative stress, among others, cannot be discarded.

The development of hypotrophic inward remodeling was detected in MRA from SOLF mice. Vascular remodeling is another major vascular alteration that has been described in obesity. However, studies performed in DIO models [7], as well as in genetic models of obesity [42,43,44] and obese humans [45], have reported the development of hypertrophic outward remodeling instead of hypotrophic inward remodeling and independently of an elevation in blood pressure. Intriguingly, the vascular remodeling observed in arteries from SOLF mice has been also described in several models of hypertension such as the ouabain-induce hypertensive rat [46] or the MWF rat, also exhibiting albuminuria [20], and could be associated to enhanced collagen deposition compromising vascular distensibility and favoring the development of hypertension [46]. In this regard, a shift toward the left in the stress–strain relationship together with enhanced β-values were detected in SOLF, UOLF, and HF mice, thus evidencing increased arterial stiffness in the three experimental groups. Increasing evidence supports the association between obesity and arterial stiffness. In fact, numerous studies performed in obese humans have revealed an increase in pulse wave velocity (PWV) [36,47,48,49], indicative of systemic stiffness. Similarly, studies in models of genetic or DIO rodents have also described an increase in intrinsic arterial stiffness either in conduit [50,51] or in resistance arteries [7].

To better understand the mechanisms involved in the impairment of vascular distensibility induced by SOLF, UOLF, and HF diet, we assessed the content and organization of the two main proteins contained in the vascular extracellular matrix, which are responsible for vessels elasticity (elastin) and resistance to changes in blood pressure (collagen). In our model, no changes were detected in elastin content in the EEL or IEL nor in the fenestrae area and number, with the exception of the HF group that exhibited a reduction in fenestrae number. In this direction, we have previously showed a reduction in fenestrae area and number associated with increased arterial stiffness in mice fed a 45% HF diet for 32 weeks [7]. However, although it is known that IEL organization is more relevant than elastin content in the contribution to arterial stiffening [7,21], the present data discard a role for elastin in arterial stiffness induced by SOLF and UOLF, at least when administered for a short period of time (8 weeks). In contrast, a significant increase in total collagen content was detected in SOLF, UOLF, and HF mice. Numerous studies performed in different models of hypertension have stated a clear relationship between alterations in collagen turnover in favor of type I/III collagen synthesis and the development of arterial stiffness in resistance arteries from both obese humans and rodents [45,52,53]. Accordingly, we have previously reported enhanced deposition of type I collagen by long-term HF feeding [7] in murine MRA. Similar results were found in small mesenteric arteries from C57Bl6 mice fed a HF diet for 16 weeks [54] as well as in conduit arteries from a Wistar rat DIO model [55].

Together these results demonstrate that SOLF and UOLF favor the development of vascular alterations including endothelial dysfunction and arterial stiffness due to enhanced collagen deposition. Moreover, this study highlights the fact that the SOLF diet seems to be even more deleterious than the UOLF or even than commercial HF diets, since hypotrophic inward remodeling was also detected in this group.

In light of these findings, it is necessary to reassess the convenience of a massive use of palm or even HOSO in food industry and to identify the possible protective factors in oils such as olive oil in order to find potential healthier replacements. Nevertheless, and considering the high content of SFA and MUFA included in SOLF and UOLF, respectively, it would be of interest to elucidate the potential impact of these fatty acids when administered at lower doses.

## Data Availability

The data presented in this study are available on request from the corresponding author.
